# Emerging roles of the Protein Phosphatase 1 (PP1) in the context of viral infections

**DOI:** 10.1186/s12964-023-01468-8

**Published:** 2024-01-24

**Authors:** Pedro O. Corda, Mathieu Bollen, Daniela Ribeiro, Margarida Fardilha

**Affiliations:** 1 Institute of Biomedicine (iBiMED), Department of Medical Sciences, University of Aveiro, Aveiro, Portugal; 2 Department of Cellular and Molecular Medicine, Laboratory of Biosignaling & Therapeutics, Katholieke Universiteit Leuven, Louvain, Belgium

**Keywords:** Protein Phosphatase 1 (PP1), Viral infections, Virus-host interactions, Host-based target, Antiviral response

## Abstract

Protein Phosphatase 1 (PP1) is a major serine/threonine phosphatase in eukaryotes, participating in several cellular processes and metabolic pathways. Due to their low substrate specificity, PP1’s catalytic subunits do not exist as free entities but instead bind to Regulatory Interactors of Protein Phosphatase One (RIPPO), which regulate PP1’s substrate specificity and subcellular localization. Most RIPPOs bind to PP1 through combinations of short linear motifs (4–12 residues), forming highly specific PP1 holoenzymes. These PP1-binding motifs may, hence, represent attractive targets for the development of specific drugs that interfere with a subset of PP1 holoenzymes. Several viruses exploit the host cell protein (de)phosphorylation machinery to ensure efficient virus particle formation and propagation. While the role of many host cell kinases in viral life cycles has been extensively studied, the targeting of phosphatases by viral proteins has been studied in less detail. Here, we compile and review what is known concerning the role of PP1 in the context of viral infections and discuss how it may constitute a putative host-based target for the development of novel antiviral strategies.

## Introduction

Viruses are completely dependent on the host cell machinery [1] and manipulate diverse physiologic and metabolic host pathways to favour infection and ensure the proper formation of new infectious viral particles. Numerous studies have reported that protein phosphorylation is crucial for several steps of the life cycle of different viruses and that specific host protein kinases are hijacked by viruses to promote infection [2–4]. In fact, these proteins have been pinpointed as potential host-directed targets for the development of antiviral therapeutics (reviewed by García-Cárceles et al. [2]). While the role of host kinases has been extensively studied in the last decades, the role of host phosphatases in the context of viral infections is still poorly understood.

Protein phosphatase 1 (PP1), a member of the phosphoprotein phosphatase (PPP) family, catalyses an important fraction of protein Ser/Thr dephosphorylation events in eukaryotic cells [5]. This phosphatase is involved in the regulation of several cellular processes such as the cell cycle, transcription, protein synthesis, and apoptosis [6–9]. In mammalian cells, the PP1 catalytic subunit (PP1c) is encoded by three distinct genes that encode three isoforms—PP1α, PP1β/δ, and PP1γ – which are ubiquitously expressed in all tissues [10]. The PP1c isoforms have a nearly identical catalytic core (~ 90%) and mainly differ in their amino (N)- and carboxy (C)- terminal extremities [7]. All PP1c isoforms have poor subtract specificity, hence, no free PP1c pools are expected to exist in cells, to prevent uncontrolled and aberrant dephosphorylation events [11]. However, PP1 counteracts the activity of over 100 kinases, which is explained by the interaction with regulatory subunits, known as Regulatory Interactors of Protein Phosphatase One (RIPPO), that tightly control the substrate selectivity, localization, and activity of PP1c [6–8, 11, 12]. Currently, about 200 structurally unrelated vertebrate RIPPOs are known [13], enabling cells to generate a huge diversity of functionally distinct PP1 holoenzymes. Most RIPPOs have short linear motifs (SliMs) that mediate binding to PP1. The most common PP1-binding SLiMs are the so-called RVxF, SILK, MyPhoNE, and ΦΦ motifs [14, 15] that dock to surface grooves on the globular catalytic core of PP1c [12, 14, 16]. In addition, some RIPPOs interact with isoform-specific residues in the N- or C-termini of PP1c, accounting for their fairly selective binding to one PP1 isoform [7, 17, 18]. Most RIPPOs have multiple PP1-binding motifs that, together, create a high-affinity interaction interface with PP1c [14].

In recent years, some studies have reported that different viruses are able to hijack PP1 and subvert its activity to favour infection. Here, we review the current knowledge on the role of PP1 within different viruses’ life cycles, discuss its importance for the antiviral immune response, and suggest PP1 and its related mechanisms as potential host-based targets for the development of new antiviral therapies.

## PP1 in viral infections

### PP1 promotes Tat-induced transcription in human immunodeficiency virus infection

**Fig. 1:**
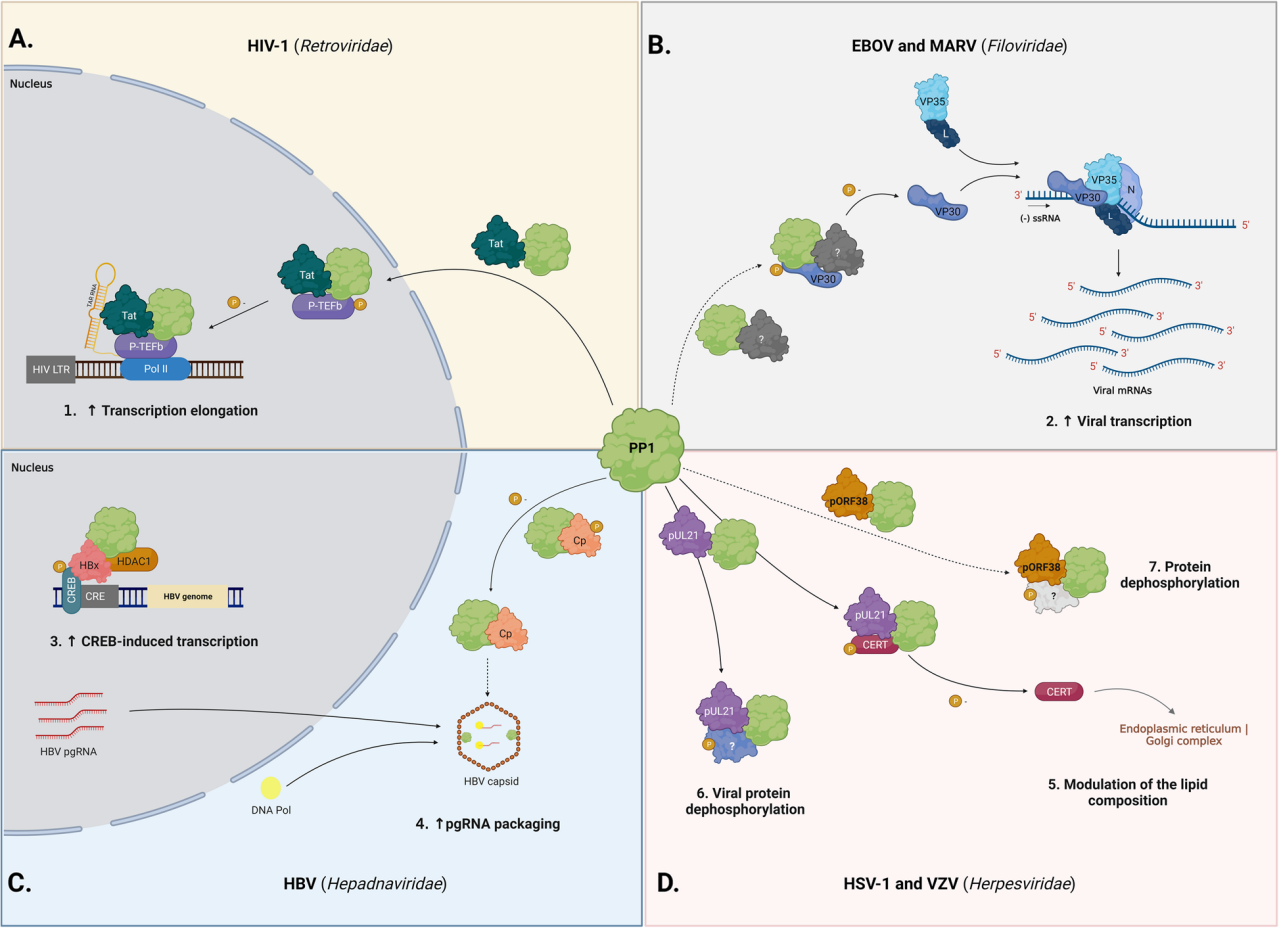
Schematic representation of the interplay between PP1 and different viruses. **A** HIV-1 Tat recruits PP1 for nuclear translocation, thereby promoting the P-TEFb dephosphorylation at the CDK9 Thr186 and enhanced transcription elongation of the viral genome. **B** EBOV and MARV VP30 are substrates of PP1 and their dephosphorylation shifts the RNA polymerase complex activity towards viral transcription. Since VP30 proteins are devoid of PP1-binding motifs, the PP1:VP30 interaction appears to be mediated by an unknown RIPPO. **C** In CREB-induced transcription, PP1 is recruited by HDAC1 to promote CREB dephosphorylation and inhibition. HBx binds to the CREB and interacts with the PP1:HDAC1 complex, to prevent CREB dephosphorylation. Thus, HBx promotes CREB-mediated HBV transcription. Also, PP1 dephosphorylates HBV Cp and it is encapsulated together with HBV pgRNA. **D** HSV-1 pUL21 and VZV pORF38 recruit PP1 to induce dephosphorylation of other viral or host proteins. During HSV-1 infection, the PP1:pUL21 complex dephosphorylates CERT, which contributes to host lipid traffic between the endoplasmic reticulum and the Golgi complex. For all figures, dash lines represent PP1 interactions that are not fully understood. Figure created with BioRender.com

The human immunodeficiency virus (HIV) is an enveloped retrovirus from the *Retroviridae* family and its genome is composed of two copies of positive-sense single-stranded RNA. It is classified into two subtypes (HIV-1 and HIV-2), of which HIV-1 is the most prevalent and pathogenic [19]. Among HIV-1 encoded proteins, Tat is a potent transactivator expressed early in infection and has a crucial role in transcriptional activity increment. Tat promotes transcription initiation through interactions with Sp1 elements in the HIV-1 promotor [20], and transcriptional elongation through the recruitment of the host positive transcriptional elongation factor b (P-TEFb) to the transactivation response region (TAR) of the nascent viral mRNA [21]. Moreover, Tat promotes the formation of a super-elongation complex through the recruitment of other elongation factors and co-activators to the HIV-1 promotor [22, 23]. Earlier studies hypothesized a possible involvement of PP1 in Tat-induced transcription [24–27]. It was observed that the overexpression of the nuclear inhibitor of PP1 (NIPP1) led to a total block of Tat-induced transcription but had a weak impact on HIV-1 basal transcription [25, 26, 28]. The simultaneous co-expression of PP1γ and NIPP1 rescued the Tat-induced transcription [25, 28], whereas the PP1γ dead-mutant co-expression failed this rescue [27, 28]. Subsequent analyses showed an interaction between PP1γ and Tat, as well as their co-localization in the nucleus [27]. Tat possesses a consensus RVxF motif (^35^QVCF^38^) and the mutation of this PP1-binding SLiM impaired the PP1:Tat interaction, inhibited the PP1α redistribution into the nucleolus, and blocked Tat-induced transcription [27]. Together, these observations indicate that Tat acts as a viral RIPPO and enhances viral transcription elongation through dephosphorylation by PP1. But how does PP1 mediate the Tat-induced transcription? As described above, Tat recruits the transcription factor P-TEFb, a multimeric complex that includes cyclin-dependent kinase 9 (CDK9):cyclin-T1. This Ser/Thr kinase phosphorylates the C-terminal domain (CTD) of the largest subunit of RNA polymerase II (Pol II) [29, 30]. CDK9 activity is required in the early elongation phase to convert Pol II into a full elongation-competent polymerase (reviewed by Egloff [31]). When fully activated, P-TEFb is only composed of CDK9:Cyclin T1 and phosphorylated at CDK9 Thr186 [32–34]. This phosphorylation allows the interaction of CDK9 with 7SK small nuclear RNA (7SK snRNA) and hexamethylene bisacetamide-inducible protein (HEXIM1) that, together with La-related Protein 7 (LAPR7) and 7SK methylphosphate capping enzyme (MePCE), inhibit the P-TEFb complex [31, 35, 36]. P-TEFb is released from inhibition through dephosphorylation of CDK9 at Thr186 by PP1 [35, 37, 38]. During HIV-1 infection, Tat recruits P-TEFb to the TAR of the nascent viral mRNA and releases P-TEFb from its inhibitory complex through competition with HEXIM1 and 7SK snRNA [31, 39–41]. It has been suggested that Tat shuttles PP1 to the nucleus, where it dephosphorylates CDK9 Thr186 [27, 42]. The overexpression of NIPP1 or its central PP1-anchoring domain (cdNIPP1) resulted in increased levels of the CDK9 Thr186 phosphorylation [26, 28], supporting the proposed mechanism. Further research also implicated PP1 in CDK9 dephosphorylation at Ser175, promoting the up-regulation of the HIV-1 transcription [43, 44]. Together, these studies demonstrated that Tat acts as a viral RIPPO, inducing PP1 nuclear translocation and enhancing the HIV transcription elongation (Fig. 1A).

### PP1 fosters different activities of the RNA polymerase complex of Ebola and Marburg viruses

Filoviruses (*Filoviridae* family), including Ebola (EBOV) and Marburg (MARV) viruses, are enveloped non-segmented negative-sense singled stranded RNA viruses, responsible for viral haemorrhagic fevers and a high mortality rate in humans [45–48]. The EBOV RNA polymerase complex, formed by the large subunit of polymerase L, its cofactor virion protein (VP) 35, and the nucleoprotein (NP), is involved in viral RNA transcription and replication [49]. The transcriptional activator VP30 is also required, being a crucial regulator that switches the polymerase complex activity from replication to transcription [50]. The VP30 activity is regulated by its RNA binding capacity and phosphorylation state [51–53]. Dephosphorylated VP30 has a higher affinity for RNA and VP35, promoting transcription [51, 54]. On the other hand, phosphorylated VP30 has increased affinity for NP, thereby shifting the polymerase complex activity towards replication [52, 54]. The VP30 dephosphorylation is regulated by the host phosphatases PP1 and protein phosphatase 2A (PP2A) [55]. PP1 inhibition by cdNIPP1 overexpression led to increased levels of VP30 phosphorylation, suggesting that VP30 is a PP1 substrate [56]. However, when PP1 was knocked down by shRNA, there was neither an increase in VP30 phosphorylation nor a shift of RNA polymerase complex activity to replication [56]. This appeared to be mediated by the splicing factor that interacts with PQBP-1 and PP1 (SIPP1) upregulation, a RIPPO that shuttles PP1 from the nucleus to the cytoplasm [57], thereby promoting VP30 dephosphorylation and viral RNA transcription [56]. While phosphorylated EBOV VP30 shifts RNA polymerase activity from transcription to replication [50], phosphorylated MARV VP30 acts as a transcription repressor [58]. Although it was initially suggested that MARV VP30 was not required for viral transcription [59, 60], later studies proposed a key role of this protein in MARV transcription [58, 61]. The current hypothesis postulates that phosphorylated VP30 sequesters VP35 and NP, preventing MARV transcription [58]. Indeed, PP1 inhibition increased VP30 phosphorylation and its interaction with VP35 and NP, resulting in decreased MARV transcription [58].

It is not yet known how PP1 is recruited to dephosphorylate VP30 from EBOV or MARV since both VP30 lack a consensus PP1-binding SLiM. Recently, it was shown that EBOV NP possesses a conserved PP2A-binding motif that is necessary for PP2A recruitment and VP30 dephosphorylation [62]. An unknown RIPPO (either cellular or viral) may be involved in the recruitment of PP1 by filoviruses to promote VP30 dephosphorylation (Fig. 1B). Further research is needed to clarify the PP1 recruitment mechanism by EBOV and MARV.

### PP1 inhibits the hepatitis B virus CREB-induced transcription but promotes its genome packaging

Hepatitis B virus (HBV) is a small enveloped hepatotropic DNA virus from the *Hepadnaviridae* family and is responsible for acute and chronic infections worldwide [63]. The HBV X protein (HBx) is a multifunctional viral protein that modulates the expression of several cellular and viral genes through interactions with host transcriptional factors and promotes the evasion of different host signaling pathways [64]. Among the transcriptional factors, HBx binds to the cAMP response element binding protein (CREB), activating the transcription of the CREB-targeted genes and HBV DNA [64–66]. CREB activation requires its phosphorylation at Ser133, allowing the recruitment of CREB-binding protein (CBP)/p300 to promote transcription [67, 68]. CREB activity is reversed through dephosphorylation by PP1, following their recruitment by histone deacetylase 1 (HDAC1) [69, 70]. Upon HBV infection, HBx enables CREB-induced transcription through inhibition of its dephosphorylation by PP1 [65]. Though it was reported that HBx interacts with PP1:HDAC1, it does not disrupt the complex [65], suggesting that HBx sequesters this complex and avoids the CREB dephosphorylation (Fig. 1C).

Recently, PP1 was implicated in the dephosphorylation of HBV core protein (Cp), the main component of the HBV nucleocapsid [71], where pre-genomic RNA (pgRNA) is packaged and converted into viral DNA by DNA polymerase (DNA Pol) [72, 73]. The Cp CTD is composed of eight conserved Ser and Thr residues which are dynamically phosphorylated during virus infection [74, 75]. Phosphorylated Cp was observed intracellularly and in empty nucleocapsids, whereas unphosphorylated Cp was detected in mature nucleocapsids and in secreted pgRNA-containing virions [71, 76–78]. Cp dephosphorylation by PP1 appears to be necessary for pgRNA and DNA Pol encapsulation [71, 77, 79]. Indeed, the knockdown of PP1 by siRNA was associated with increased Cp phosphorylation levels and decreased pgRNA packaging [71]. Hu et al*.* also reported that PP1α and PP1β are co-packaged in pgRNA-containing nucleocapsids (Fig. 1C) [71]. A previous study reported the co-packaging of cyclin-dependent kinase 2 (CDK2), which plays a critical role in releasing HBV DNA from the nucleocapsid through the phosphorylation of the Cp N- and C-termini [80]. Noteworthy, PP1 is inhibited by CDK2 phosphorylation [81], suggesting that *a posteriori* PP1 inhibition is required for pgRNA release.

### PP1 is recruited by Herpes Simplex virus 1 pUL21 to promote dephosphorylation of viral and host proteins

Herpesviruses are large enveloped double-strand DNA viruses that can remain in a dormant, latent state in host cells [82, 83]. Herpes Simplex virus (HSV) is an alpha-herpesvirus that infects epithelial cells and establishes latency in neurons [82, 83]. The HSV-1 tegument protein pUL21 was recently described as a PP1 interactor that regulates the phosphorylation status of viral and cellular proteins [84]. In early infection, pUL21 promotes the transport of the HSV capsid to the nucleus [85, 86], whereas in late infection pUL21 is required for the nuclear egress of the HSV capsids, and to promote the glycosylation of the viral glycoprotein gE [85, 87, 88]. Co-immunoprecipitation assays showed an interaction between pUL21, all three PP1 isoforms, and the ceramide transport protein (CERT) [84]. Dephosphorylated CERT participates in the ceramide exchange and lipid traffic between the endoplasmic reticulum and the Golgi complex [89, 90]. pUL21 acts as a bridge between PP1 and CERT, promoting CERT dephosphorylation. Consequently, dephosphorylated CERT modulates the cellular lipid composition of the endoplasmic reticulum and Golgi complex and/or the post-Golgi protein traffic [84, 91]. Nevertheless, the HSV-1 replication was not significantly altered when pUL21-mediated CERT dephosphorylation was abolished in keratinocytes and epithelial cells [91]. The Varicella-zoster virus (VZV), another alpha-herpesvirus, possesses an HSV-1 pUL21 homologue—the pORF38 protein – that also binds to PP1 but does not bind to CERT, suggesting that CERT recruitment is HSV-1-specific [84]. A full understanding of the role of pUL21 in HSV-1 infection and the CERT pathway modulation requires further investigation (Fig. 1D). Ma and colleagues also demonstrate that PP1 is an interactor of the porcine pseudorabies virus (PRV) pUL21, another orthologue of the HSV pUL21 [92]. Although the functional interaction between PRV pUL21 and PP1 was not explored, these results suggest that the binding of alpha-herpesviruses to PP1 is phylogenetically conserved.

Interestingly, pUL21 is devoid of a canonical PP1-binding SLiM but has a motif in the linker region, named Twenty-one Recruitment Of Protein Phosphatase One (TROPPO), that is conserved among the alpha herpesviruses (including VZV pORF38 and PRV pUL21) and has the consensus sequence φ-S-x-F-V-Q-[VI]-[KR]-x-I, where φ is a hydrophobic residue and x any amino acid (pUL21: ^239^VSEFVQVKHI^248^) [84]. Mutations in pUL21 Phe242 and Val243 impaired PP1 binding and significantly decreased viral replication [84]. Yet, HSV pUL21 mutants showed a remarkable capacity for adaption in that they recovered their replication potential through mutations in the pUS3 gene, a viral kinase involved in HSV nuclear egress [93]. Benedyk et al*.* reported that these pUS3 mutations reduced stability and kinase activity, suggesting that pUL21 may counteract pUS3 kinase through PP1 recruitment for the same substrates (e.g. pUL31) in the later phases of infection (Fig. 1D) [84].

## PP1 plays important roles in the host cell response to viral infections

### Viral proteins recruit PP1 to maintain translation through dephosphorylation of eIF2α

**Fig. 2:**
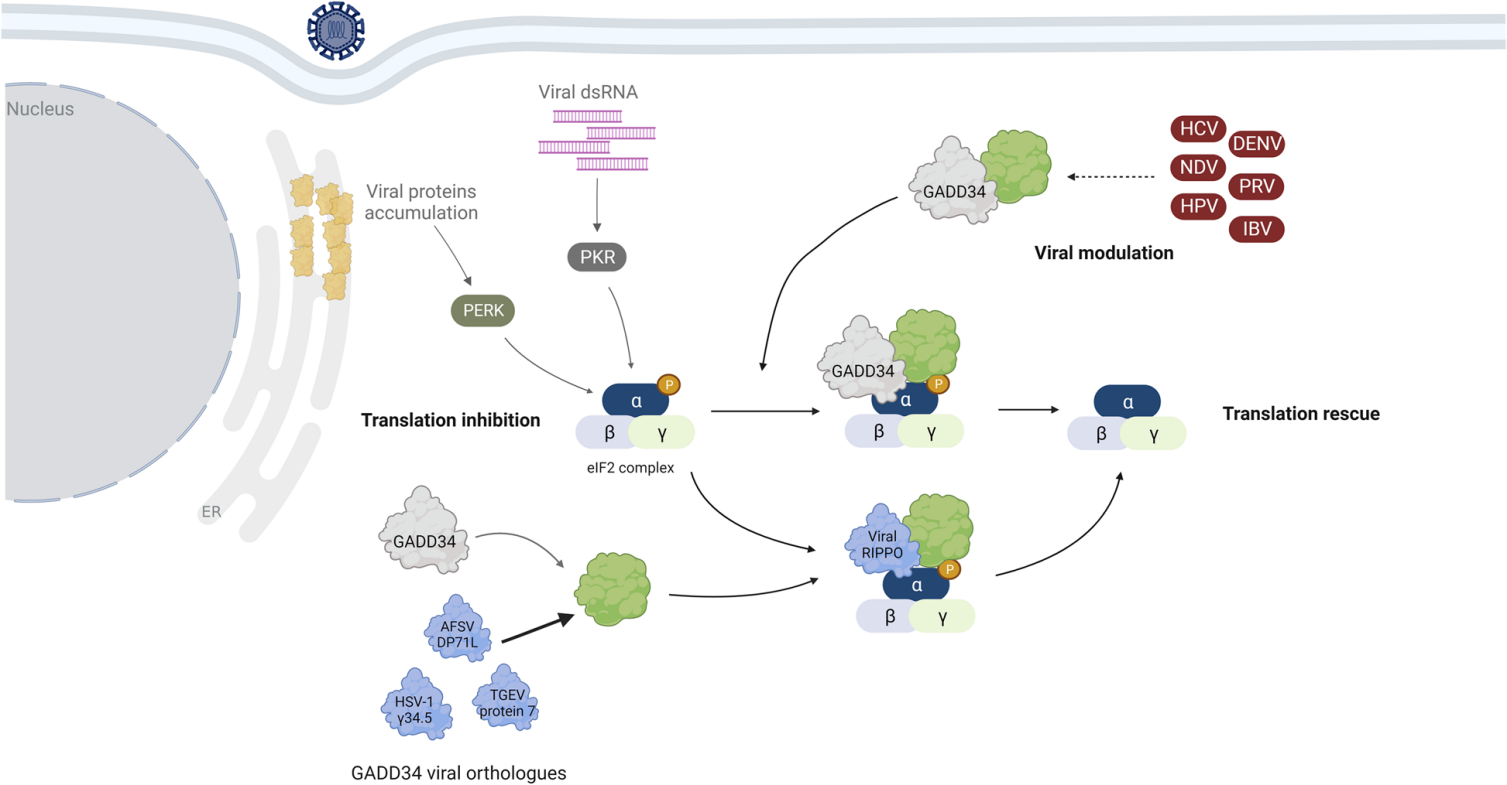
PP1 is recruited by some viruses to rescue host translation activity. dsRNA sensing and viral protein accumulation in ER activates the PKR and PERK, respectively, culminating in eIF2α phosphorylation at Ser51 and host cell translation shut-off. This process is reversed by the PP1:GADD34 holoenzyme. HSV-1 γ34.5, ASFV DP71L, and TGEV protein 7 are viral orthologues of GADD34 and interact with PP1 to promote eIF2α dephosphorylation and protein synthesis rescue. Some viruses – DENV, HCV, NDV, HPV, PRV, and IBV –modulate the activity of host-cell PP1:GADD34. Figure created with BioRender.com

Upon viral infection, the production of double-strand RNA (dsRNA) induces activation of the dsRNA-dependent protein kinase R (PKR), which phosphorylates the eukaryotic initiation factor 2 subunit α (eIF2α) at Ser51 and blocks the translation initiation [94, 95]. Moreover, the accumulation of viral proteins in the endoplasmic reticulum (ER) induces the activation of the protein kinase R-like ER kinase (PERK), which also phosphorylates eIF2α at Ser51 [96]. Yet, this translational block is reversed through eIF2α dephosphorylation by PP1. Two RIPPOs, Growth arrest and DNA damage-inducible protein (GADD34, also known as PPP1R15A) and Protein Phosphatase 1 regulatory subunit 15B (PPP1R15B, also known as CREP) recruit PP1 to promote eIF2α dephosphorylation [97, 98]. However, viruses evolved mechanisms to prevent eIF2α phosphorylation by PKR and PERK or to promote eIF2α dephosphorylation through the recruitment of PP1 (Fig. 2) or PP2A [99].

In HSV-1-infected cells, the neurovirulence factor γ34.5 is mainly responsible for keeping eIF2α dephosphorylated [100]. The C-terminus of γ34.5 has a primary structure similar to the C-terminal domain of GADD34 [101]. This γ34.5 region contains a validated RVxF motif (HSV-1: ^192^RVRF^195^) that mediates the recruitment of PP1α (Fig. 2) [102, 103]. Mutations of this motif abolished eIF2α dephosphorylation, impaired HSV-1 replication, and increased the sensitivity to interferon response [102, 104, 105]. The γ34.5 C-terminus also possesses an Ala-Arg-rich motif that directly binds to eIF2α [106–109], acting as a bridge between PP1 and the phosphorylated eIF2α. Furthermore, Meng et al. reported that HSV-1 γ34.5 enables the shuttling of ribosome biogenesis protein NOP53 from the nucleus to the cytoplasm, which was suggested to facilitate PP1α recruitment by γ34.5 [110]. Despite NOP53 seeming to be necessary for efficient HSV-1 viral replication [110], its role in the PP1 recruitment by the γ34.5 is still unknown. The African swine fever virus (ASFV) DP71L protein is another GADD34 viral orthologue [101]. The ASFV encodes a DP71L long form (184 aa) and a short form (70–72 aa), both possessing a RVxF motif: ^124^KVYF^127^ and ^14^KHVRF^18^, respectively [101, 111]. ASFV infection also induces a translation shut-off, being this response counter-acted by the PP1 recruitment mediated by the DP71L and subsequent dephosphorylation of the eIF2α (Fig. 2) [112–114]. In addition to the RVxF motif, DP71L also has a LSAVL motif (long form: position 169–173; short form: position 57–61) that is required to bridge the interaction between PP1 and eIF2α [111]. Noteworthy, the mutant ASFV ∆DP71L still possesses the capacity to induce eIF2α dephosphorylation, suggesting that other ASFV proteins may regulate the eFI2α phosphorylation [114].

The transmissible gastroenteritis virus (TGEV) protein 7 interacts with PP1 through a RVxF motif (^58^RVIF^61^) that is conserved in other α-coronaviruses’ (α-CoVs) protein 7, including the canine coronavirus (CcoV), porcine respiratory coronavirus (PRCV), and feline infectious peritonitis (FIPV) [115]. Co-immunoprecipitation assays showed that TGEV protein 7 interacts with both PP1 and eIF2α [115]. Compared to cells infected with wildtype TGEV, cells infected with TGEV lacking protein 7 (TGEV ∆7) had higher levels of phosphorylated eIF2α and showed premature protein synthesis inhibition, widespread apoptosis, and exacerbated immune response [115, 116]. TGEV ∆7-infected pigs presented increased tissue damage and proinflammatory response [115, 116]. Although protein 7 is not essential for TGEV replication, it appears to attenuate the virulence of the infection in both in vitro and in vivo models. Similarly, in infected mice with severe acute respiratory syndrome coronavirus (SARS-CoV), a beta coronavirus (β-CoV), the loss of the PP1 inhibitor Kepi was also associated with increased virus pathogenicity [117]. Hence, PP1 activity appears to be required to avoid severe pathological damage during TGEV and SARS-CoV infections.

Other viruses such as dengue virus (DENV) [118], hepatitis C virus (HCV) [119], the New Castle disease virus (NDV) [120], human papillomavirus (HPV) [121], PRV [122, 123], and infectious bronchitis virus (IBV) [124] appears to maintain protein translation and low eIF2α phosphorylation levels through stimulatory interactions with the host-cell PP1:GADD34 holoenzyme (Fig. 2). In addition, some of those viruses increase GADD34 expression during late infection, which contributes to increased dephosphorylation of eIF2α through the PP1:GADD34 [118, 120, 121, 123, 124]. Nevertheless, the GADD34 up-regulation should be carefully analysed since it is a downstream effect of the eIF2α phosphorylation (through the ATF4/Chop pathway) even in other stress-related conditions [125]. Therefore, the GADD34 increment may not be a specific phenomenon caused by a virus. Further research is required for a better understanding of the PP1:GADD34 holoenzyme role in those viral infections.

### PP1 in Rig-I-like receptors’ activation and its modulation by measles virus and α-coronaviruses

RIG-I-like receptors (RLRs) are a family of cytosolic RNA sensors for the host defense against viral infections and include three members: retinoic-acid inducible gene I (RIG-I), melanoma differentiation association gene 5 (MDA5), and Laboratory of genetics and physiology 2 (LGP2) [126]. Although RIG-I and MDA5 recognize different viral RNA molecules, they are structurally similar and possess two caspase activation and recruitment domains (CARDs) at the N-terminus, which mediate the downstream signal transduction [126, 127]. Upon recognition of viral RNA, RIG-I or MDA5 oligomerizes, allowing the subsequent interaction with the mitochondrial antiviral-signalling protein (MAVS) at mitochondria and peroxisomes [128, 129]. MAVS is responsible for the induction of the subsequent signalling leading to the production of interferons (IFNs), interferon-stimulated genes (ISGs), and other cytokines [130]. To induce an INF response, MAVS recruits the tumour necrosis factor receptor-associated factor 3 (TRAF3) and TRAF family member-associated NF-κB activator (TANK). Subsequently, the TRAF3/TANK complex activates the IκB Kinase ε (IKKε) and TANK-binding kinase 1 (TBK1), two kinases responsible for the phosphorylation of the interferon regulatory factors (IRF) 3 and 7 [131]. Then, IRF3 and IRF7 are translocated to the nucleus and induce the expression of type I INF [130].

**Fig. 3:**
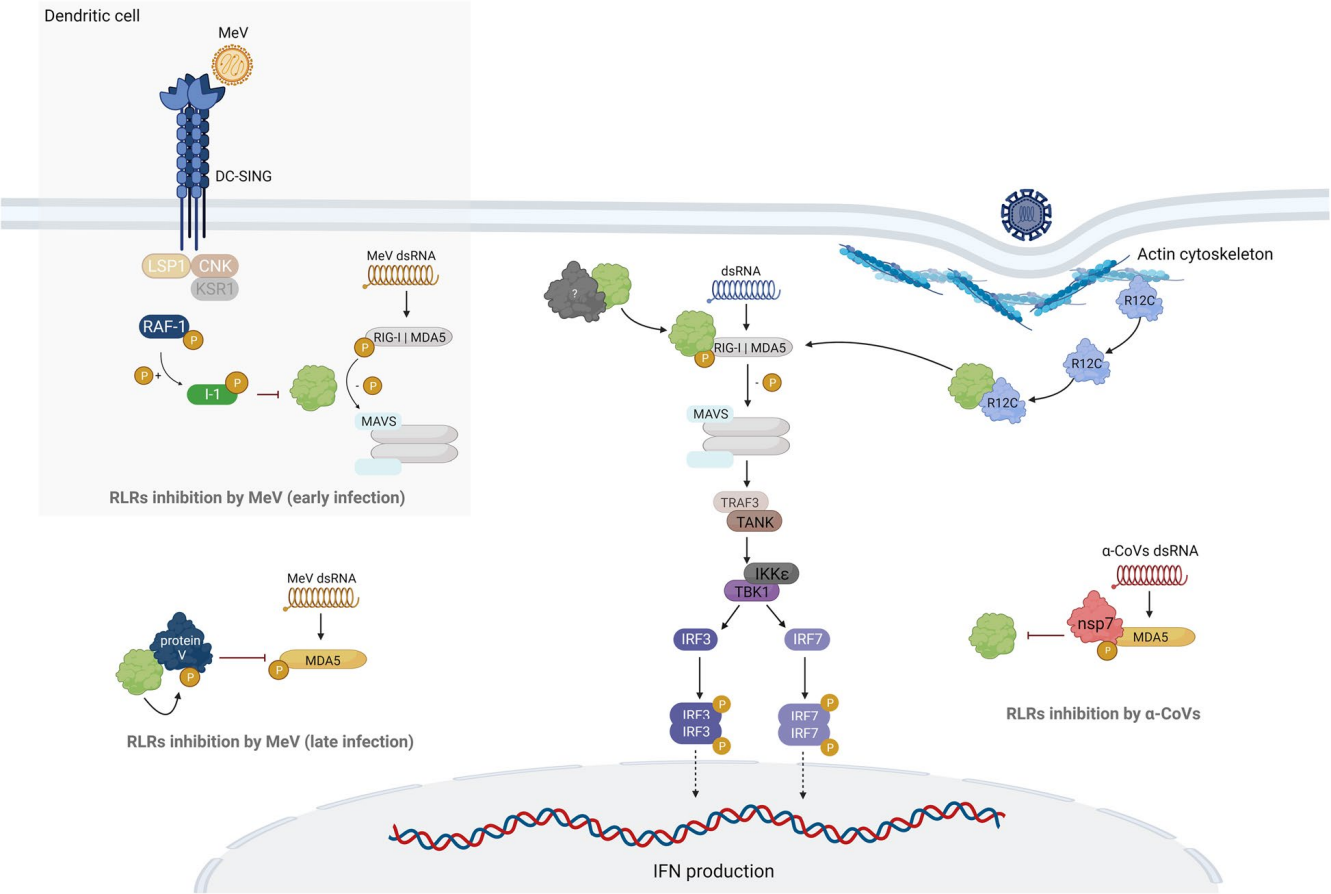
PP1 is required for the RLRs activation, and its activity is modulated by MeV and α-CoVs. Upon dsRNA sensing, PP1 is recruited (by unknown RIPPOs) to dephosphorylate RIG-I/MDA5 and promote their activation. Recently, it was shown that as a consequence of the cytoskeleton disturbance, R12C recruits PP1 to dephosphorylate RIG-I and MDA5. In the early infection of dendritic cells, MeV interacts with the DC-SING receptor leading to the activation of Raf-1 kinase. Raf1 phosphorylate inhibitor 1 (I-1), a potent PP1 inhibitor, avoids RIG-I/MDA5 activation. In late MeV infection, protein V prevents MDA5 dephosphorylation by acting as a PP1-substrate. In α-CoV infections, the nsp7 binds to MDA5 N-terminus and blocks its dephosphorylation by PP1. Figure created with BioRender.com

RLR activation is a tightly regulated process and different studies have demonstrated that it highly depends on protein phosphorylation [132]. In resting cells, RIG-I and MDA5 exist in an inactive conformational state where Thr and Ser residues in CARDs are phosphorylated (RIG-I: Ser8 and Thr170; MDA5: Ser88) [133–135]. Upon infection, the RIG-I/MDA5 CARDs are rapidly dephosphorylated by PP1α/γ (Fig. 3), followed by K63-linked polyubiquitination that triggers MAVS-dependent downstream signalling [135, 136]. RIG-I and MDA5 possess two PP1-binding SLiMs: a F-x-x-R/K-x-R/K motif at the CARD (RIG-I: ^94^FKKIEK^99^; MDA5: ^18^FRARVK^23^), and a RVxF motif at the helicase region (RIG-I: ^292^KVVFF^296^; MDA5: ^397^KISF^400^) [135]. While both MDA5 PP1-binding SLiMs are required for the interaction with PP1, only the RIG-I F-x-x-R/K-x-R/K motif appears to be necessary for this interaction [135]. However, the PP1 recruitment mechanism to dephosphorylate RLRs after viral dsRNA recognition is not fully understood. Recently, Acharya et al*.* reported that the Protein phosphatase 1 regulatory subunit 12C (PPP1R12C or R12C) mediates the RIG-I/MDA5 dephosphorylation by PP1 [137]. R12C participates in the actomyosin cytoskeleton dynamic regulation as part of myosin phosphatase complex [138]. Upon viral infection, the disturbance of the actin cytoskeleton induces the subcellular redistribution of R12C and the formation of the R12C:PP1:RLR complex, which dephosphorylates RIG-I/MDA5 and promotes the downstream signalling (Fig. 3) [137]. Thus, R12C acts as an upstream regulator of the RLR pathway, recruiting PP1 to RIG-I/MDA5 and promoting their activation. Still, further investigation is required to unravel other RIPPOs involved in the RIG-I/MDA5 dephosphorylation mediated by PP1.

The measles virus (MeV), a paramyxovirus, evolved several mechanisms to escape the IFN response [139–141], two of which are related to PP1 targeting to suppress the RIG-I/MDA5 dephosphorylation [142]. In the first mechanism, the MeV interacts with the C-type lectin DC-SIGN receptor in the dendritic cell (DC) surface and induces the phosphorylation and activation of protein kinase Raf1. Raf1 then phosphorylates inhibitor 1 (I-1), a specific inhibitor of the PP1:GADD34 holoenzyme [143]. The phosphorylated I-1 blocks RIG-I/MDA5 dephosphorylation and subsequent activation (Fig. 3) [143]. The second mechanism involves the MeV protein V, an accessory protein that is crucial to the suppression of the host IFN response [144, 145]. At the late infection stage, protein V antagonizes the MDA5 dephosphorylation by PP1α/γ and serves as a putative PP1 substrate, maintaining PP1 away from MDA5 (Fig. 3) [146]. The MeV protein V has a consensus RVxF motif in the C-terminus region (^288^RIWY^291^). Mutation or deletion of this motif reduced the PP1 binding to MeV protein V and increased MDA5 dephosphorylation [146]. It was also reported that the protein V of the Nipah virus (NiV), another paramyxovirus, interacts with PP1 [146]. However, contrary to MeV protein V, NiV protein V is devoid of PP1-binding SliMs [146]. This observation may suggest that PP1 recruitment by paramyxoviruses is required for their survival. Together, these studies showed that MeV avoids RLR activation by preventing both RIG-I and MDA5 dephosphorylation in a Raf1-dependent manner during early infection, and the MDA5 dephosphorylation in a protein V-dependent manner in the late infection [142, 143, 146].

Several studies have reported that various coronaviruses encode immunomodulatory proteins capable of suppressing RLR signalling [92, 147–151]. The α-CoV porcine epidemic diarrhoea virus (PEDV) non-structural protein 7 (nsp7) inhibits the RLR pathway by preventing the MDA5 dephosphorylation [152]. The nsp7 competes directly with PP1α/γ for binding to MDA5 and thereby prevents MDA5 Ser828 dephosphorylation and subsequent MAVS signalling [152, 153]. This mechanism also applies to other α-CoVs that encode orthologues of the PEDV nsp7 such as TGEV, swine acute diarrhoea syndrome coronavirus (SADS-CoV), and feline coronavirus (FCoV) [152].

### PP1 is a negative regulator of the Toll-like receptor (TLR) pathway and is recruited by HSV-1 and enteroviruses to modulate TLR signaling

**Fig. 4:**
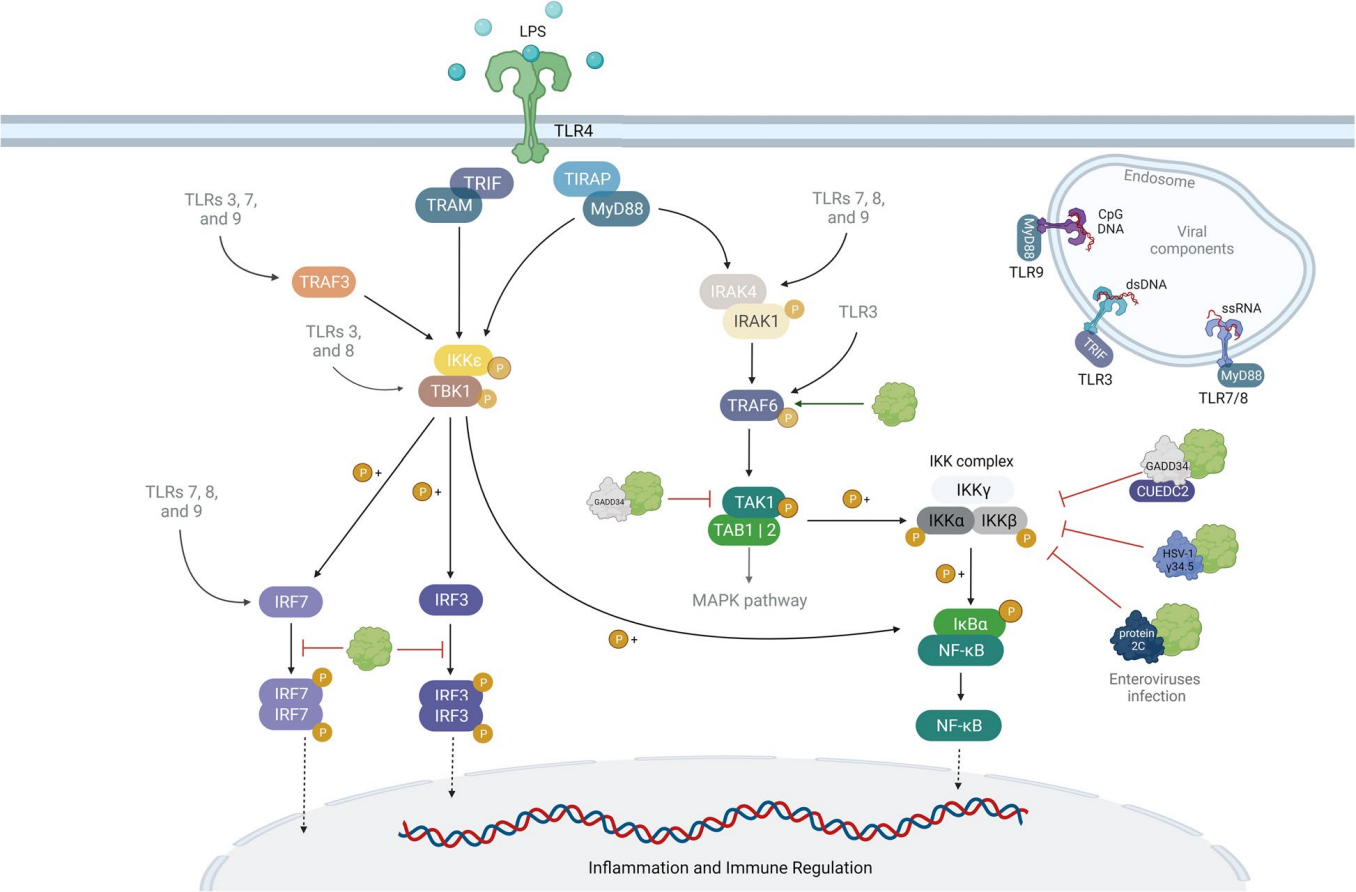
PP1 is a negative regulator of the TLR pathway and is recruited by viruses to impair TLR signaling. PP1:GADD34 holoenzyme counteracts the activation of TAK1 and IKK, two crucial kinases in the TLR cascade. In resting cells, IKK is maintained in an inactive state by the PP1:GADD34:CUEDC2 complex. Upon viral infection, PP1 avoids the excessive activation of IRF3 and IRF7 and limits IFN-α/β expression. HSV-1 γ34.5 and enteroviruses (EV71, PV, CVA, and CVB) protein 2C recruit PP1 to dephosphorylate IKKβ and inhibit NF-κB activation. Figure created with BioRender.com

Toll-like receptors (TLR) are transmembrane glycoproteins located at the plasma membrane or endosomal membranes and sense a vast number of pathogen- and damage-associated molecular patterns [154]. Upon activation, TLRs trigger a downstream signalling cascade that culminates in the activation of transcription factors, interferon regulatory factors (IRFs), and nuclear factor kappa-light chain enhancer of activated B cells (NF-κB) [155]. Among the 10 known human TLRs, TLR3, TLR7, TLR8, and TLR9 are involved in the recognition of viral nucleic acids [156]. TLR7, TLR8, and TLR9 signal through the myeloid differentiation primary response protein 88 (MyD88) adaptor to activate the NF-κB or mitogen-activated protein kinase (MAPK) pathways, resulting in the expression of proinflammatory cytokines [156]. TLR7-9 also promotes the IFN response through the activation of IRF-5 and IRF-7. TLR3 signals through the TIR domain-containing adaptor protein inducing IFN-β (TRF) adaptor, leading to the activation of both IRF-3 and NF-κB signaling [157]. Phosphorylation by various kinases is crucial for TLR activation [158]. PP1 acts as a negative regulator of the TLR pathway by dephosphorylation of different mediators (Fig. 4).

Three main Ser/Thr kinase families are conserved elements of TLR signaling: interleukin-1 receptor kinases (IRAKs), the tumor growth factor β-activated kinase (TAK1), and inhibitors of NF-κβ (IκB) kinase (IKK) complex [159]. TAK1 is activated by ubiquitination and phosphorylation at Thr184, Thr187, and Ser412 [160, 161]. PP1:GADD34 holoenzyme dephosphorylates TAK1 at Ser412, thereby preventing downstream NF-κB and MAPK activation [162]. Although all three PP1 isoforms can interact directly with TAK1, Ser412 dephosphorylation is dependent on GADD34, which possesses a TAK1-binding domain [162]. The IKK complex is formed by two Ser/Thr kinases (IKKα and IKKβ) and a regulatory scaffolding protein NF-kβ essential modifier (NEMO, also known as IKKγ) [163, 164]. In resting cells, IKK is sequestered in the cytoplasm by the PP1:GADD34:CUE domain–containing protein 2 (CUEDC2) complex that maintains IKK in a non-phosphorylated, inactive state [165]. In TNF-stimulated mouse embryonic fibroblasts, IKK is released from its inhibitory complex by tumor necrosis factor receptor-associated factor 2 (TRAF2) and is phosphorylated by the TNF receptor. After activation, the IKK is released from the TNF-receptor signaling complex and is available to interact with CUEDC2. GADD34 mediates the interaction between PP1 and CUEDC2 and promotes IKK dephosphorylation and inactivation [165]. A similar phenomenon was observed in LPS-stimulated cells (a TLR signaling inductor), being the IKK released from its inhibitory complex by TRAF6 [165]. Together, these results are indicative that PP1 negatively regulates specific steps of TLR signaling.

Upon viral recognition by TLRs, IRF3, and IRF7 are activated through phosphorylation of key Ser/Thr residues, and subsequently induce IFN production [166–168]. In NDV-infected cells, inhibition of PP1 enhanced IRF7 activation and IFN-α production, and impaired NDV replication [169]. PP1 binds to IRF7, which possesses two RVxF motifs (^11^RVLF^14^ and ^408^RVFF^411^), and reduces its activation through dephosphorylation [169]. Like IRF7, IRF3 possesses a RVxF motif (^213^RQVF^216^) that is necessary for the PP1 binding [170]. In turn, PP1 prevents full IRF3 activation, decreasing the TLR- and RLR-mediated IFN-β expression [170].The human enterovirus EV71 – the causative agent of hand, foot, and mouth disease – inhibits IKKβ and suppresses the NF-κβ signaling pathway [171]. The EV71 protein 2C forms a complex with PP1 and IKKβ, promoting IKKβ dephosphorylation and inactivation (Fig. 4). The EV71 protein 2C possesses three RVxF motifs (motif 1: ^16^KGLEG^20^; motif 2: ^27^KFIDW^31^; motif 3: ^43^KVEF^46^) that appear to be required for PP1 recruitment [171]. Three other enteroviruses – poliovirus (PV), coxsackievirus A (CVA), and coxsackievirus B (CVB) – also inhibit IKKβ dephosphorylation through PP1 recruitment by their protein 2C ortholog [171]. In HSV-1-infected DCs, γ34.5 binds to IKKα/β via the N-terminus and recruits PP1 via the C-terminus, preventing the IKKβ phosphorylation and NF-κβ activation (Fig. 4) [172]. This negatively impacts DC maturation, where the TLR-4/NF-κβ pathway is activated to promote the expression of inflammatory and co-stimulatory molecules that are essential for maturation [173]. Collectively, these studies highlight how viruses can subvert the regulatory role of PP1 in TLR signaling to promote their escape from the host immune response.

Although most studies highlighted the role of PP1 as a negative regulator of the TLR pathway, Opaluch et al*.* proposed that PP1 may positively regulate TLR signaling (Fig. 4) [174]. Upon TLR4/5/7 stimulation, PP1γ dephosphorylates TRAF6 and promotes downstream signaling events including NF-κB activation [174]. Yet, the full mechanism behind PP1:TRAF6 interaction and regulation remains poorly understood.

### PP1-mediated dephosphorylation of myxovirus resistance 2 (MX2) is required for the innate response against HIV-1

The innate immune response mobilizes an antiviral state that stimulates the production of IFNs, which induces the expression of IFN-stimulated genes that participate in antiviral responses against viral pathogens [175]. One of the IFN-stimulated genes is MX2 (also named MXB), a GTPase related to MX1 that presents a potent inhibitory activity against HIV-1 [176–179], HSV-1, and HSV-2 replication [180, 181]. In HIV-1 infection, MX2 blocks the nuclear entry of the viral genome by binding to the HIV-1 capsid, which precludes viral DNA accumulation and integration into host chromosomes [182, 183]. Betancor et al*.* showed that the N-terminal domain (NTD) of MX2 interacts with PP1β:myosin phosphatase target subunit 1 (MYPT1) holoenzyme [184]. PP1 inhibition or depletion increased the MX2 NTD phosphorylation (Ser 14, 17, and 18), and reduced the ability of MX2 to bind to the HIV-1 capsid [184]. This suggests that PP1β regulates the antiviral activity of MX2. The NTD of MX2 has a F-x-x-R/K-x-R/K motif (^8^WPYRRR^13^) that also contributes to the binding of the PP1β:MYPT1 [184]. Together, these findings suggest that MX2 is regulated by phosphorylation, and its IFN-dependent activation may also induce MX2 dephosphorylation [184].

## PP1 as potential target for antiviral therapies

Classical antiviral therapies are focused on the inhibition of viral processes by targeting viral proteins [185–187]. However, this strategy often fails due to viral adaptation, which leads to the emergence of drug-resistant mutants [188]. During viral infections, several interactions between viral and host proteins are established and maintained to facilitate virus surveillance and propagation. Therefore, targeting host-based mechanisms became an efficient alternative strategy to fight viruses, allowing the design of molecules that interfere with host proteins hijacked by them. Such molecules are expected to have a broad spectrum activity and to decrease viral drug resistance [189, 190].

For a long time, phosphatases were considered unattractive targets for the development of drug therapies [191]. The design of selective modulators for PP1’s active site is highly challenging due to its great conservation within the PPP family [192, 193]. However, PP1 holoenzymes can be selectively targeted by interference with PP1:RIPPO interactions [16, 194]. One effective approach is the design and synthesis of PP1-disrupting peptides that allow the disruption of PP1 holoenzymes’ interactions and the modulation of certain processes [195–198]. In this sense, the PP1-drug targeting landscape has been explored to fight viral infections. Through in silico modulation, a set of molecules was designed based on the HIV-1 Tat RVxF motif, leading to the synthesis of 1,2,3,4-tetrahydracridine (1H4) that decreases the interaction between PP1 and Tat, and significantly inhibits HIV-1 transcription and replication [199]. Based on the 1H4 structure, a library of compounds was designed to improve HIV-1 inhibition, of which compound 1E7-03 showed a strong HIV-1 inhibition capacity with a lower IC_50_ (five-fold compared to 1H4) [200]. 1E7-03 was also the first PP1-small target molecule that significatively inhibited HIV-1 transcription in mice [201]. While 1E7-03 had strong in vivo inhibitory capacity, it had lower metabolic stability being converted into two products (DP1 and DP3) that bind PP1 but present low cell penetration capacity [201]. Further structural improvement of 1E7-03 led to the synthesis of the compound HU-1a, which has an overall superior HIV-1 inhibitory capacity as well as improved metabolic stability [202]. The 1E7-03 treatment diminished the replication of the Rift Valley fever virus (RVFV) and the Venezuelan equine encephalitis virus (VEEV) [203, 204], suggesting that PP1 also plays a role in these viral infections. Indeed, Carey et al*.* showed that PP1 interacts with the VEEV capsid and regulates at least two phases of viral replication: the early post-entry and late viral assembly [204]. The same authors also reported that other alphaviruses – including Eastern Equine Encephalitis Virus (EEEV), West Equine Encephalitis Virus (WEEV), Sindbis virus (SINV), and Chikungunya virus (CHIKV) – also presented impaired viral replication after 1E7-03 treatment [204]. After EBOV infection, 1E7-03 inhibited VP30 dephosphorylation and prevented the EBOV transcription [56]. Surprisingly, 1E7-03 also strongly inhibited EBOV replication in a dose-dependent manner, which may result from the transcription/replication imbalance promoted by high levels of phosphorylated VP30 [56]. Two 1E7-03 analogues—1E7-07 and C31 – also prevented VP30 dephosphorylation and viral transcription [205, 206]. In MARV-infected cells, 1E7-03 increased VP30 phosphorylation and decreased MARV transcription and replication [58].

In latent HIV-infected cells, one major concern is that integrated proviruses can become activated and produce viral proteins when antiretroviral therapy is interrupted or drug resistance emerges [207–209]. Eradication of latent HIV-1 is challenging since all clinically approved anti-HIV drugs are ineffective unless the viral transcription is activated. Tyagi et al*.* discovered a sulfonamide-containing compound, named small-molecule activator of PP1 (SMAPP1) that induced HIV-1 transcription in latent HIV-1 infected cells [210]. SMAPP1 is a suitable compound for the kick-and-kill approach, where the HIV-1 is reactivated and then fought through an antiretroviral combination [210].

## Concluding remarks

During viral infections, there is an extensive network of interactions occurring between viral and host proteins, not only to promote viral replication but also to increase the host antiviral response. As a multifaceted player that participates in several crucial processes for cell survival and maintenance, it does not come as a surprise that PP1 stands out as one of these modulated host cell factors.

In this review, we discussed the interplay between viral proteins and host-cell PP1 in the context of different viral infections. We highlight that viruses interact with PP1 via two mechanisms: (i) some viruses encode a viral RIPPO (HIV Tat, HBV HBx, HSV pUL21) that interacts with PP1 and promotes the dephosphorylation of host and/or viral proteins; or (ii) viruses encode a protein that is a PP1-substrate (EBOV/MARV VP30, HBV Cp, MeV protein V). Both mechanisms involve the recruitment of PP1 to promote viral propagation. Although this topic has been explored for about two decades, only a few studies present detailed mechanistic data on how PP1 is recruited and how its activity is crucial for virus infection. So far, the role of PP1 in HIV-1 infection is the best characterized. Importantly, some viruses such as filoviruses, herpesviruses, α-CoVs, and enteroviruses appear to retain PP1-binding motifs, suggesting that PP1-recruitment may be crucial for those viruses’ adaption across their evolution.

PP1 is also involved in the regulation of different host antiviral responses and, therefore, some viruses modulate PP1 activity to evade host defence mechanisms. In fact, HSV-1 and ASFV encode viral RIPPOs that are orthologs of host-cell GADD34 and bind to PP1 to induce the eIF2α dephosphorylation. Also, TGEV protein 7 mimics the GADD34 role to direct PP1 to reestablish the translation activity. Regarding RLRs and TLRs pathways, despite most kinases involved in Ser/Thr phosphorylation of these pathways have been widely studied, the phosphatases that counteract their activity remain poorly investigated. One aspect that needs to be clarified is how PP1 is recruited to dephosphorylate the different receptors and whether there are other RIPPOs (still unknown) that promote and direct PP1 activity to these substrates upon receptor activation. Intriguingly, PP1’s role in the cGAS-STING pathway, which is activated by cytosolic DNA (self and invaded viral or microbial) needs to be further explored in the context of viral infection. One study reported that PP1 regulates the cGAS dephosphorylation during mitosis [211], suggesting that PP1 may play a regulatory role in this pathway.

As stated in the previous section, the design and synthesis of compounds that disturb the interaction between PP1 and viral RIPPOs may be an effective strategy to fight viral infections. PP1-based antiviral therapies appear to have a broad spectrum of viral inhibitory activity, as shown with the 1E7-03 compound. Further and more detailed studies are necessary to complement the knowledge on how different viruses modulate and take advantage of PP1 activity and how this can be exploited to develop novel antiviral therapies.
